# Pamiparib dose escalation in Chinese patients with non‐mucinous high‐grade ovarian cancer or advanced triple‐negative breast cancer

**DOI:** 10.1002/cam4.3575

**Published:** 2020-10-31

**Authors:** Binghe Xu, Yongmei Yin, Mei Dong, Yan Song, Wei Li, Xiang Huang, Tongshan Wang, Jing He, Xiyan Mu, Li Li, Song Mu, Wa Zhang, Miao Li

**Affiliations:** ^1^ National Cancer Center/Cancer Hospital Chinese Academy of Medical Sciences and Peking Union Medical College Beijing China; ^2^ The First Affiliated Hospital of Nanjing Medical University Nanjing China; ^3^ BeiGene (Shanghai) Co., Ltd. Shanghai China; ^4^ BeiGene (Beijing) Co., Ltd. Beijing China; ^5^ BeiGene USA, Inc. San Mateo CA USA

**Keywords:** BGB‐290, high‐grade ovarian cancer, pamiparib, PARP inhibitor, triple‐negative breast cancer

## Abstract

**Background:**

The recommended phase 2 dose (RP2D) of pamiparib, an investigational PARP1/2 inhibitor, was established as 60 mg twice daily (BID) in a first‐in‐human (FIH) study (NCT02361723).

**Methods:**

Chinese patients with advanced non‐mucinous high‐grade ovarian cancer (HGOC) or triple‐negative breast cancer (TNBC) whose disease either progressed despite standard therapy, or for which there is no standard therapy were enrolled in the dose‐escalation (DE) portion of a phase 1/2 study (NCT03333915). The primary endpoint was safety/tolerability; secondary objectives were pharmacokinetics and antitumor activity. *BRCA1*/*2* mutation status was retrospectively evaluated.

**Results:**

Nine HGOC and six TNBC patients (N = 15; n = 4, 20 mg; n = 4, 40 mg; n = 7, 60 mg) were enrolled; as of 30 September 2019, one HGOC patient remained on treatment. Seven patients (n = 5, HGOC; n = 2, TNBC) had germline *BRCA1*/*2* mutation (*gBRCA* ^mut^); all HGOC patients were resistant/refractory to platinum. Asthenia and nausea (n = 12 each) were the most common treatment‐related adverse events (TRAEs). Decreased hemoglobin was the most common grade 3 TRAE (n = 3); no grade ≥4 AEs were observed. No dose‐limiting toxicities (DLTs) were reported. Pamiparib plasma exposure was similar to exposure observed in the FIH study after a single‐dose administration, albeit slightly higher at steady state. Among 13 RECIST‐evaluable patients, two with HGOC (*gBRCA* ^mut^, n = 1) achieved a confirmed partial response and six with HGOC (*gBRCA* ^mut^, n = 4) achieved stable disease; all TNBC RECIST‐evaluable patients (n = 5) reported progressive disease.

**Conclusions:**

Pamiparib was generally well tolerated in Chinese patients, with durable responses observed in patients with HGOC. Based on these results, pamiparib 60 mg BID was confirmed as the RP2D.

## Graphical abstract

Pamiparib (an investigational PARP1/2 inhibitor) 60 mg BID was confirmed as the recommended phase 2 dose in Chinese patients with advanced non‐mucinous high‐grade ovarian cancer (HGOC) or triple‐negative breast cancer whose disease progressed despite standard therapy, or for which there is no standard therapy. Pamiparib 60 mg BID was generally well tolerated with asthenia and nausea being the most commonly reported treatment‐related adverse events. Clinical durable responses were observed among patients with HGOC, all of whom were resistant/refractory to platinum chemotherapy and most with *gBRCA* ^mut^.

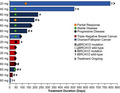

## 1 INTRODUCTION

Poly (ADP‐ribose) polymerase 1 and 2 (PARP1/2) enzymes are associated with nuclear process regulations, including the repair of DNA, genomic stability, and programmed cell death.^1^ The primary function of PARP1/2 enzymes is to detect DNA breaks (single‐strand) and target them for repair.^2^ Inhibiting PARP enzymes ultimately leads to the conversion of single‐strand breaks to double‐strand breaks, resulting in genomic instability and apoptosis.^2^ Repair of double‐strand DNA breaks normally occurs through homologous recombination in the presence of tumor suppressor proteins BRCA1 and BRCA2.^2^ Within BRCA‐deficient cells, homologous recombination is impaired, leading to genomic instability, which may favor tumorigenesis.

Small molecule inhibitors of PARP (PARPi) represent a new class of therapeutic agents being used to treat patients with different types of cancers with genomic instability, including tumors associated with *BRCA1/2* mutations.^3^, ^4^ The mechanism of PARP inhibition is currently under investigation. Inhibitors of PARP have been found to bind directly to and inhibit the activity of PARP enzymes, thus preventing DNA repair and trapping PARP–DNA complexes at the site of DNA damage.^5^, ^6^ Agents that inhibit PARP are particularly effective treatments for tumors with homologous recombination deficiency (HRD) because they cannot accurately repair the DNA damage.

Pamiparib (BGB‐290) is an investigational selective inhibitor of PARP1/2 that has been shown to have PARP–DNA complex trapping capabilities and brain penetration.^7^ Pamiparib suppressed PARP activity in patient‐acquired xenografts of glioblastoma multiforme and small cell lung cancer.^7^, ^8^, ^9^ Additionally, pamiparib was found to be 10‐fold more potent than olaparib^8^ and was able to potentiate the effects of temozolomide.^7^, ^9^ Preliminary results of the first‐in‐human (FIH) study (NCT02361723) demonstrated that pamiparib was generally well tolerated and showed antitumor activity, notably in patients with non‐mucinous high‐grade ovarian cancer (HGOC).^10^, ^11^, ^12^ The FIH study also established the recommended phase 2 dose (RP2D) of pamiparib as 60 mg orally (PO) twice daily (BID) and demonstrated that pamiparib exposure is linear up to 80 mg BID, which has been determined as the maximum tolerated dose (MTD).^10^, ^12^ Here, we present results from the phase 1 component of an ongoing phase 1/2 study in Chinese patients with advanced HGOC or advanced triple‐negative breast cancer (TNBC) (NCT03333915); results of the ongoing phase 2 component of the study are forthcoming.

## 2 METHODS

### 2.1 Study design

**Figure 1:**
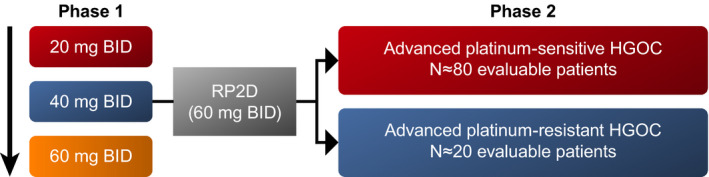
Study Design. Abbreviations: BID, twice daily; HGOC, high‐grade ovarian cancer; RP2D, recommended phase 2 dose

This phase 1/2, open‐label, multicenter study assessed the safety and antitumor activity of pamiparib in Chinese patients ≥18 years with advanced solid tumors whose disease progressed despite standard therapy or for which there is no standard therapy. Phase 1 was a dose‐escalation study, with a 3 + 3 design, that evaluated the single‐agent safety/tolerability, pharmacokinetic (PK) profile, and preliminary antitumor activity of pamiparib and confirmed the RP2D in Chinese patients (Figure 1). The phase 2 dose‐expansion component of this study is investigating the single‐agent antitumor activity and safety/tolerability of pamiparib.

In phase 1, patients received pamiparib at 20, 40, and 60 mg BID dose levels. The starting dose for pamiparib (20 mg BID) was selected as one‐third of the RP2D (60 mg BID) and one‐fourth of the MTD (80 mg BID), which were determined in the FIH study. Results of the FIH study showed a favorable risk‐benefit profile for each dosage.^10^, ^12^ A 23‐day initial treatment cycle (dose‐limiting assessment window) of each dose level consisted of a single administration of pamiparib on Day 1 followed by a treatment‐free period on Day 2 and a 21‐day period of BID administration from Day 3 to Day 23. Safety evaluations, including dose‐limiting toxicities (DLTs), of ≥3 patients completing one cycle of treatment at a specific dose level were required before proceeding to the next cohort with a higher dose level. Patients could continue the study treatment in 21‐day cycles and subsequent cycles at the discretion of the investigator.

Phase 2 enrolled platinum‐sensitive HGOC patients (disease progression occurring ≥6 months after last platinum treatment [Cohort 1]) or platinum‐resistant HGOC patients (Cohort 2). Patients with either known deleterious or suspected deleterious germline *BRCA* mutation (*gBRCA* ^mut^) who previously received at least two lines of standard chemotherapy were eligible for enrollment.

### 2.2 Phase 1 study population

Phase 1 enrolled patients with histologically or cytologically confirmed locally advanced or metastatic HGOC, including fallopian and primary peritoneal cancer, or TNBC for which no effective standard therapy was available. *BRCA1/2* mutations were not required, but enrichment of this patient population was preferred. Patients with *gBRCA* ^mut^ were retrospectively identified by central testing. When available, archival tumor tissues were collected to assess tumor *BRCA* (*tBRCA*) status. Patients were defined as platinum refractory if disease progression occurred during their last platinum treatment and as platinum resistant if disease progression occurred less than 6 months after their last platinum treatment. Patients were required to have measurable disease per Response Evaluation Criteria in Solid Tumors (RECIST) v1.1,^13^ an Eastern Cooperative Oncology Group (ECOG) performance status of 0–1, and adequate organ function. Patients were not permitted to have received chemotherapy, biologic therapy, immunotherapy, investigational agent, anticancer Chinese medicine or anticancer herbal remedies, or to have undergone radiotherapy for any cause within 14 days of the first dose of study drug. Patients who had untreated and/or active brain metastases, or had previously received therapies targeting PARP, were excluded from the study.

### 2.3 Phase 1 endpoints and assessments

The primary objective in phase 1 was to evaluate the safety and tolerability of pamiparib. Safety and tolerability endpoint assessments were based on monitoring of treatment‐emergent adverse events (AEs), in addition to vital signs, electrocardiograms, physical examinations, and clinical laboratory results. The incidence and severity of all AEs and incidence of serious AEs were assessed according to the National Cancer Institute‐Common Terminology Criteria for Adverse Events v4.03.

Secondary objectives were assessment of PK parameters and antitumor activity. Blood samples for the PK analysis were collected at Cycle 1 on Days 1, 2, and 3. Pharmacokinetic sampling was performed at Cycle 1 on Day 1 (predose and at 0.5, 1, 2, 4, 6, 9, and 12 hours postdose), Day 2, Day 3, and Day 10 (predose and at 0.5, 1, 2, 4, 6, 9, and 12 hours postdose); then at Cycles 2 and 3 on Day 1 (predose and at 2 hours postdose). Across all patients, assessments of antitumor activity included objective response rate (ORR), duration of response (DoR), durable clinical benefit rate (CBR, defined as the percent of patients achieving complete response [CR], partial response [PR], or stable disease [SD] of ≥24 weeks), and progression‐free survival (PFS) per investigator assessment based on RECIST v1.1. Tumor assessments were performed by the investigators at screening, every 6 weeks after the first dose of pamiparib in the 1st year, and every 12 weeks thereafter until progression, per RECIST v1.1. Patients completed the study at the time of their safety follow‐up visit; therefore, radiological assessment of disease progression was not performed after the safety follow‐up visit.

Among eligible patients with HGOC, carcinoma antigen 125 (CA‐125) response rate per Gynecologic Cancer Intergroup was also assessed. Patients with HGOC had CA‐125 tested in a local laboratory at baseline, every 6 weeks in the 1st year, and then, every 12 weeks thereafter. CA‐125 responses must have been confirmed and maintained for at least 28 days. Germline and *tBRCA1/2* mutations were analyzed as predictive biomarkers of antitumor activity and resistance to pamiparib. *BRCA* mutations were assessed using next‐generation sequencing, performed by BGI Genomics Co., Ltd, using the germline (*gBRCA*) or somatic (*tBRCA*) Hereditary Breast and Ovarian Cancer Test.

### 2.4 Analysis populations

Approximately 14 to 18 evaluable patients were expected to enroll in the phase 1 part of the study. The safety population included all patients who received ≥1 dose of pamiparib. The PK population included all patients for whom valid pamiparib PK parameters could be estimated. Antitumor activity per RECIST v1.1 was assessed in all evaluable patients. Patients were considered response evaluable if they had measurable disease at baseline per RECIST v1.1 and had ≥1 post‐baseline tumor assessment, unless treatment had been discontinued due to clinical progression or death prior to tumor assessment. The population evaluable for CA‐125 response included patients in the safety population with baseline CA‐125 ≥2 x upper limit of normal.

### 2.5 Statistical methods

Descriptive statistics were used to summarize all study data. Continuous variables included the number of non‐missing observations, mean, standard deviation, median, minimum, and maximum. Categorical variables were summarized by their frequency and percentage. Progression‐free survival was estimated using the Kaplan–Meier method and the corresponding 95% confidence interval (CI) was constructed using Greenwood's formula. Standard PK parameters (i.e., area under the plasma concentration‐time curve [AUC] from time of drug administration; maximum plasma concentration [C_max_]; time to C_max_; elimination half‐life [t_1/2_]; apparent clearance; and apparent volume of distribution during terminal phase) were estimated and derived using standard non‐compartmental methods.

The study was conducted in accordance with Good Clinical Practice and all applicable regulatory requirements, including the Declaration of Helsinki. All patients provided written informed consent.

## 3 RESULTS

### 3.1 Disposition, demographic, and baseline disease characteristics

Here, we present the results from the 15 Chinese female patients (HGOC, n = 9; TNBC, n = 6) who were enrolled in the phase 1 portion of this study (Table 1). Overall median age was 49 years (range: 32–70), and 73% of patients had previously received at least three lines of therapy. A total of seven patients were confirmed as having a *gBRCA* ^mut^ (HGOC, n = 5; TNBC, n = 2); eight patients had g*BRCA* wild type (g*BRCA* ^WT^; HGOC, n = 4; TNBC, n = 4). A total of eight patients had qualified tumor samples available and had *tBRCA* status assessed (HGOC, n = 6; TNBC, n = 2). Five of these eight patients were confirmed to be carrying *tBRCA* mutations, all of whom also had *gBRCA* ^mut^ (HGOC, n = 3; TNBC, n = 2); the other three patients had wild‐type germline and *tBRCA*.

**Table 1:** Patient demographics and baseline characteristics

	20 mg BID (n = 4)	40 mg BID (n = 4)	60 mg BID (n = 7)	Total (N = 15)
Tumor type, n (%)				
Ovarian^a^	1 (25.0)	1 (25.0)	4 (57.1)	6 (40.0)
Fallopian^a^	1 (25.0)	1 (25.0)	1 (14.3)	3 (20.0)
Triple‐negative breast cancer	2 (50.0)	2 (50.0)	2 (28.6)	6 (40.0)
Median age, years (range)	52.5 (48–62)	47.0 (32–66)	49.0 (46–70)	49.0 (32–70)
ECOG status, n (%)				
0	0	1 (25.0)	1 (14.3)	2 (13.3)
1	4 (100.0)	3 (75.0)	6 (85.7)	13 (86.7)
Number of prior lines of therapy, n (%)				
1	0	1 (25.0)	0	1 (6.7)
2	1 (25.0)	2 (50.0)	0	3 (20.0)
3	2 (50.0)	0	2 (28.6)	4 (26.7)
≥4	1 (25.0)	1 (25.0)	5 (71.4)	7 (46.7)
*gBRCA* status, n (%)				
*BRCA1* mutation	0	1 (25.0)	4 (57.1)	5 (33.3)
*BRCA2* mutation	0	0	2 (28.6)	2 (13.3)
*BRCA* wild type	4 (100.0)	3 (75.0)	1 (14.3)	8 (53.3)
*tBRCA* status, n (%)				
*BRCA1* mutation	0	1 (25.0)	1 (14.3)	2 (13.3)
*BRCA2* mutation	0	0	1 (14.3)	1 (6.7)
Both *BRCA1* and *BRCA2* mutation	0	0	2 (28.6)	2 (13.3)
*BRCA* wild type	1 (25.0)	1 (25.0)	1 (14.3)	3 (20.0)
Unknown	3 (75.0)	2 (50.0)	2 (28.6)	7 (46.7)
Median time from initial diagnosis, years (range)	3.7 (1.0–7.6)	2.0 (0.4–3.4)	5.2 (1.4–14.1)	3.4 (0.4–14.1)

Abbreviations: BID, twice daily; *BRCA*, breast cancer susceptibility gene; ECOG, Eastern Cooperative Oncology Group; *gBRCA*, germline *BRCA*; *tBRCA*, tumor *BRCA*.

^a^ Included in HGOC population.

As of 30 September 2019, median study follow‐up was 3.5 months (range, 1.2–32.7), 14 patients had discontinued study treatment, and one HGOC patient remained on treatment (Table S1). The primary reason for discontinuation was disease progression (n = 8); other reasons for discontinuation were AEs (n = 2), investigator decision (n = 2), patient decision (n = 1), and loss to follow‐up (n = 1). Across the 15 patients, median duration of treatment was 2.5 months (range, 0.3–32.7).

### 3.2 Safety/tolerability profile

Across the pamiparib dose range (20–60 mg BID), no DLTs were reported; RP2D was confirmed as 60 mg PO BID. All 15 patients experienced ≥1 AE of any grade, with asthenia (80%), nausea (80%), decreased white blood cell count (67%), and decreased appetite (60%) being most commonly reported. A total of 10 (67%) patients experienced grade ≥3 AEs, and the most commonly reported were anemia (27%) and decreased hemoglobin (20%). All patients (100%) also experienced ≥1 AE considered related to pamiparib; asthenia and nausea (80% each) were the most commonly reported treatment‐related AEs (TRAEs) (Table 2). A total of six (40%) patients experienced ≥1 TRAE of grade 3 severity; decreased hemoglobin (n = 3) was the only grade 3 TRAE reported in >2 patients. Other grade 3 TRAEs observed included anemia, decreased neutrophil count, and decreased white blood cell count (n = 2, each); and decreased platelet count, prolonged QT interval, nausea, and vomiting (n = 1, each). No grade 4 or 5 TRAEs were observed in this population.

**Table 2:** Treatment‐related adverse events of any grade (≥20% in the total population) and grade ≥3

	20 mg BID (n = 4)		40 mg BID (n = 4)		60 mg BID (n = 7)		Total (N = 15)	
	All Grades	Grade ≥3	All Grades	Grade ≥3	All Grades	Grade ≥3	All Grades	Grade ≥3
Asthenia	3 (75.0)	0	4 (100.0)	0	5 (71.4)	0	12 (80.0)	0
Nausea	4 (100.0)	0	3 (75.0)	0	5 (71.4)	1 (14.3)	12 (80.0)	1 (7)
Decreased white blood cell count	2 (50.0)	0	3 (75.0)	0	5 (71.4)	2 (28.6)	10 (66.7)	2 (13)
Decreased appetite	3 (75.0)	0	3 (75.0)	0	3 (42.9)	0	9 (60.0)	0
Decreased neutrophil count	1 (25.0)	0	3 (75.0)	0	4 (57.1)	2 (28.6)	8 (53.3)	2 (13)
Decreased hemoglobin	0	0	1 (25.0)	0	5 (71.4)	3 (42.9)	6 (40.0)	3 (20)
Increased conjugated bilirubin	1 (25.0)	0	1 (25.0)	0	2 (28.6)	0	4 (26.7)	0
Prolonged electrocardiogram QT^a^	1 (25.0)	1 (25.0)	1 (25.0)	0	2 (28.6)	0	4 (26.7)	1 (7)
Vomiting	1 (25.0)	0	1 (25.0)	0	2 (28.6)	1 (14.3)	4 (26.7)	1 (7)
Decreased platelet count	0	0	0	0	3 (42.9)	1 (14.3)	3 (20.0)	1 (7)
Diarrhea	0	0	1 (25.0)	0	2 (28.6)	0	3 (20.0)	0
Increased GGT	1 (25.0)	0	1 (25.0)	0	1 (14.3)	0	3 (20.0)	0
Somnolence	1 (25.0)	0	1 (25.0)	0	1 (14.3)	0	3 (20.0)	0
Tachycardia	0	0	1 (25.0)	0	2 (28.6)	0	3 (20.0)	0

Data presented as n (%).

Abbreviations: BID, twice daily; GGT, gamma‐glutamyl transferase.

^a^ Three patients had QTcF 450–480 ms without clinical sequelae, and one patient had QTcF ≥500 ms (grade 3 QT prolongation).

Overall, three patients experienced a serious AE (abdominal infection, n = 1; pleural effusion, n = 1; intestinal obstruction, n = 1), none of which were considered related to study treatment. Two patients discontinued treatment as a result of serious AEs (abdominal infection, n = 1; pleural effusion, n = 1).

### 3.3 Pharmacokinetic profile of pamiparib

**Figure 2:**
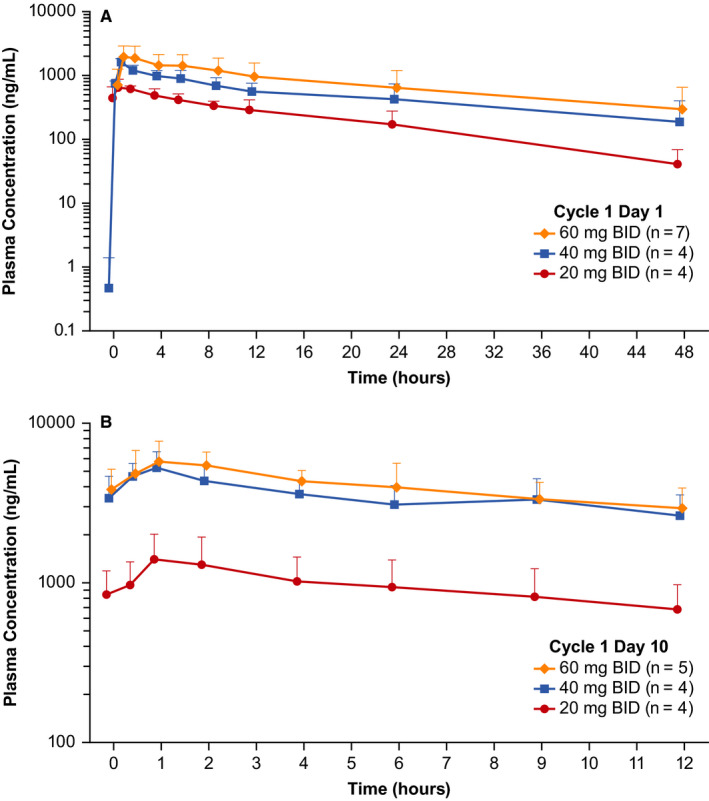
Pharmacokinetic Profile of Pamiparib After a Single Dose (A) and at Steady State (B). A, Mean concentration‐time profile of single‐dose pamiparib (Cycle 1 Day 1). B, Mean concentration‐time profile of pamiparib at steady state (Cycle 1 Day 10)

Mean pamiparib plasma concentration‐time profiles after a single dose (Cycle 1 Day 1) and at steady state (Cycle 1 Day 10) are presented in Figure 2. A summary of the PK parameters of pamiparib are presented in Table 3. Pamiparib plasma exposure (AUC and C_max_) increased near proportionally with increasing doses between 20 mg and 60 mg BID after a single dose (Table S2). At the RP2D of 60 mg BID, pamiparib is rapidly absorbed and eliminated after oral administration with median T_max_ of approximately 1 hour and t_1/2_ of approximately 14 hours; steady‐state geometric mean (%CV) AUC_0‐12_ and C_max_ were 47099.9 (30.4) ng/mL*h and 5861.3 (29.2) ng/mL, respectively. The AUC and C_max_ accumulation ratios at 60 mg BID were 3.4 and 2.6, respectively (Table 4).

**Table 3:** Summary of single‐dose and steady‐state pharmacokinetic parameters of pamiparib

	N	C_max_ (ng/ml)	T_max_ ^a^ (hr)	AUC_0‐9_ (ng/ml*h)	AUC_0‐12_ (ng/ml*h)	AUC_0‐inf_ (ng/ml*h)	t_1/2_ ^b^ (hr)	CL/F (L/hr)	Vz/F (L)
Single dose									
20 mg BID	4	718.4 (18.9)	1.01 (0.52, 3.78)	4129.9 (13.1)	5033.4 (16.6)	10240.5 (43.9)	12.14 (8.15, 18.15)	1.95 (44)	3.42 (24)
40 mg BID	4	1633.7 (12.2)	0.98 (0.97, 1.05)	8557.6 (20.1)	10364.2 (22.7)	19312.7 (61.1)	11.79 (8.74, 20.14)	2.07 (61)	3.52 (22)
60 mg BID	7	2302.5 (30.1)	1.13 (1.00, 6.08)	11895.2 (47.0)	14826.0 (47.7)	27454.4 (79.4)	13.77 (6.54, 34.91)	2.19 (79)	3.72 (33)
Steady state									
20 mg BID	4	1280.1 (70.5)	1.04 (0.92, 2.12)	8357.6 (67.7)	10317.2 (68.6)	NA	NA	NA	NA
40 mg BID	4	5213.8 (25.6)	1.10 (0.98, 1.95)	32469.1 (28.6)	40906.1 (30.2)	NA	NA	NA	NA
60 mg BID	5	5861.3 (29.2)	1.13 (0.98, 1.97)	38135.6 (29.6)	47099.9 (30.4)	NA	NA	NA	NA

Data are presented as geometric mean (geometric coefficient of variation [CV]%), unless otherwise indicated.

Single‐dose assessments were made on Cycle 1 Days 1, 2, and 3. Steady‐state assessments were made on Cycle 1 Day 10.

Abbreviations: AUC, area under the plasma concentration‐time curve from time of drug administration to time (9 or 12 hours) or to infinity; BID, twice daily; CL/F, apparent clearance; C_max_, maximum observed plasma concentration; NA, not assessed; t_1/2,_ elimination half‐life; T_max_, time to reach C_max_; Vz/F, apparent volume of distribution during terminal phase.

^a^ Data presented as median (range).

^b^ Data presented as geometric mean (range).

**Table 4:** Pamiparib accumulation rate

Parameter	Dose	n	Accumulation ratio	95% CI
AUC_0‐12_ (h*ng/mL)	20 mg BID	4	2.05	1.52‐2.77
AUC_0‐12_ (h*ng/mL)	40 mg BID	4	3.95	2.92‐5.34
AUC_0‐12_ (h*ng/mL)	60 mg BID	5	3.36	2.56‐4.40
C_max_ (ng/mL)	20 mg BID	4	1.78	1.16‐2.75
C_max_ (ng/mL)	40 mg BID	4	3.19	2.07‐4.92
C_max_ (ng/mL)	60 mg BID	5	2.62	1.78‐3.85

Abbreviations: AUC, area under the plasma concentration‐time curve from time of drug administration to time (12 hours) or to the last measurable concentration; BID, twice daily; CI, confidence interval; C_max_, maximum observed plasma concentration.

### 3.4 Antitumor activity

**Figure 3:**
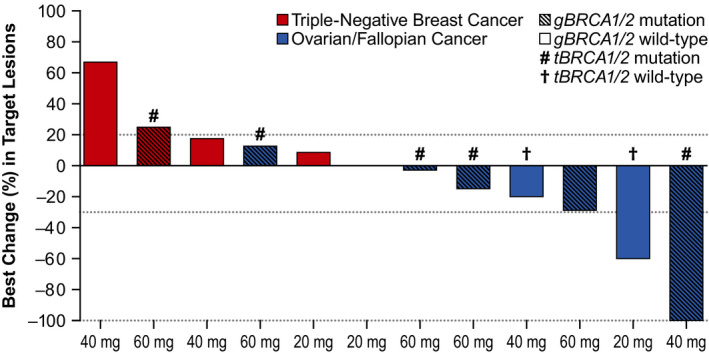
Clinical Response by Tumor Type in the RECIST Efficacy‐Evaluable Population (N = 13)^a^. ^a^Only patients who had measurable disease at baseline and at least one post‐baseline tumor assessment were included in the efficacy‐evaluable population. One patient did not have a post‐baseline assessment of target lesions and is, therefore, not included in this figure. Abbreviations: *BRCA*, breast cancer susceptibility gene; RECIST, Response Evaluation Criteria in Solid Tumors

**Figure 4:**
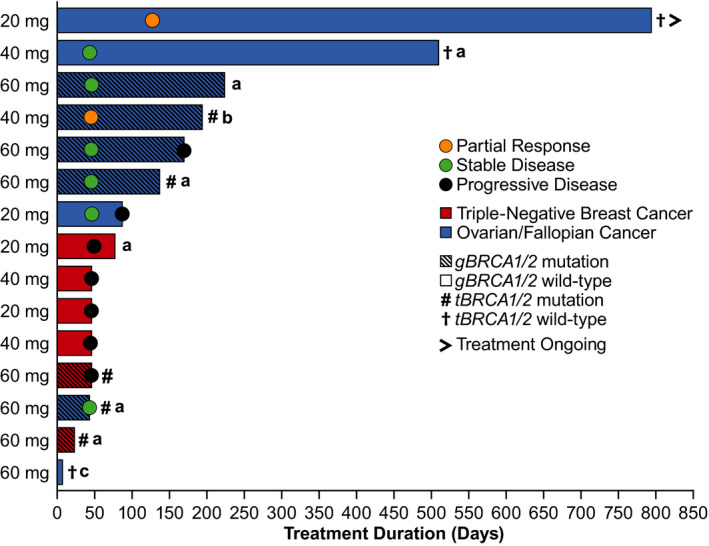
Duration of Treatment and Response by Tumor Type and *BRCA* Mutation Status (RECIST v1.1) in the Overall Population (N = 15). ^a^Discontinued due to reasons other than AE or PD; ^b^Discontinued due to AE unrelated to treatment; ^c^Discontinued due to AE that was possibly treatment related. The first dot for each bar indicates timepoint when best overall response was achieved. Abbreviations: *BRCA*, breast cancer susceptibility gene; RECIST, Response Evaluation Criteria in Solid Tumors

Of the eight RECIST‐evaluable patients with HGOC, all of whom were refractory (n = 1) or resistant (n = 7) to platinum chemotherapy, two (*gBRCA* ^mut^, n = 1; *gBRCA* ^WT^, n = 1) patients achieved a confirmed PR and six (*gBRCA* ^mut^, n = 4; *gBRCA* ^WT^, n = 2) achieved SD (Table 5; Figure 3). Across the patients with HGOC, the durable CBR was 62.5% (95% CI, 24.5–91.5) and median treatment duration was ~6 months (182 days; range: 43–995) (Figure 4). At data cut‐off, treatment was ongoing for one patient with HGOC (20 mg dose level) who achieved a PR; at data cut‐off, response duration for this patient was 27.7 months. Eight patients with HGOC were CA‐125 evaluable; one patient achieved a confirmed CR and one achieved a confirmed PR (Table S3). Median time to response (per CA‐125 criteria) was 1.45 months (range: 1.4–1.5).

**Table 5:** Best overall response by tumor type in the RECIST efficacy‐evaluable population (N = 13)^a^

	HGOC (n = 8)	TNBC (n = 5)
Best overall response, n (%)		
Complete response	0	0
Partial response	2 (25.0)	0
Stable disease	6 (75.0)	0
Progressive disease	0	5 (100.0)
Objective response rate (CR+PR), % (95% CI)	25.0 (3.2‐65.1)	0
Disease control rate (CR+PR+SD), % (95% CI)	100.0 (63.1‐100)	0
Clinical benefit rate (CR+PR+SD ≥24 weeks), % (95% CI)	62.5 (24.5‐91.5)	0

Abbreviations: CI, confidence interval; CR, complete response; HGOC, high‐grade ovarian cancer; PR, partial response; SD, stable disease; TNBC, triple‐negative breast cancer.

^a^ Only patients who had measurable disease at baseline and at least one post‐baseline tumor assessment were included in the efficacy‐evaluable population.

Best overall response for all five patients with RECIST‐evaluable TNBC was progressive disease (Table 5; Figure 3). Four of these patients were *gBRCA* ^WT^ and one harbored both germline and *tBRCA* mutations. All patients with TNBC experienced disease progression during or after their previous platinum‐based chemotherapy and all patients had distant metastases to the lung, liver, or bone at study entry.

### 3.5 Progression‐free survival

The median follow‐up of PFS for patients with HGOC was 5.6 months (95% CI: 0.03‐23.6) (Table S4). At the time of data cut‐off, two disease progressions have been collected among HGOC patients. The estimated event‐free rate at 9 months in patients with HGOC was 68.6% (95% CI: 21.3‐91.2). It should be noted that the PFS data might be incomplete for HGOC patients because more than half of the patients reached end of treatment for reasons other than disease progression; however, radiological assessment of disease progression was not performed after the safety follow‐up visit, per phase 1 study design. Among patients with TNBC (n = 6), median PFS was 1.5 months (95% CI: 1.5‐1.6), and at the time of data cut‐off, all TNBC patients with ≥1 post‐baseline tumor assessment (n = 5) had disease progression.

## 4 DISCUSSION

Development of PARPi therapy over the past decade has resulted in novel therapeutic choices for patients with ovarian and breast cancer. The phase 1 DE component of this phase 1/2 study aimed to examine the safety and tolerability, PK profile, and antitumor activity of single‐agent pamiparib as well as confirm 60 mg BID to be the RP2D in Chinese patients with advanced HGOC or TNBC.

At doses of 20 to 60 mg BID, pamiparib was generally well tolerated by patients in this study. The resulting safety and tolerability profile of pamiparib is similar to the profiles of other PARPi. The majority of AEs observed with pamiparib were generally mild‐to‐moderate in severity with asthenia and nausea being the most commonly reported AEs considered related to treatment. While most reported TRAEs were grade ≤2, decreased hemoglobin was the only grade 3 TRAE reported in more than two patients; no grade 4 or 5 TRAEs were reported.

Pamiparib was rapidly absorbed (T_max _= ~1 hr) and plasma exposure increased in a near dose‐proportional manner. Plasma exposure after administration of a single dose of pamiparib was similar to plasma exposure observed in the FIH study,^10^, ^12^ while the steady‐state exposure was slightly higher.

Confirmed and durable clinical responses were observed with pamiparib in patients with HGOC as assessed by both RECIST v1.1 and CA‐125 criteria. All RECIST‐evaluable patients with HGOC (n = 8) achieved either a PR or SD (DCR = 100%; durable CBR = 62.5%); all patients were considered refractory (n = 1) or resistant (n = 7) to platinum chemotherapy. Clinical responses were observed among patients with *gBRCA* ^WT^ as well as in patients with disease resistant/refractory to platinum‐based chemotherapy. As of 30 September 2019, one patient with HGOC refractory to platinum‐based chemotherapy and *gBRCA* ^WT^ from the 20 mg DE cohort still remains on treatment with a best response of PR.

The phase 1 portion of this study confirmed pamiparib 60 mg BID as the RP2D in Chinese patients. This confirmation was based on the observation that the safety/tolerability and PK profiles observed in the 15 Chinese patients were generally consistent with that from the FIH study,^10^, ^11^, ^12^ and that no DLTs or serious AEs considered related to pamiparib were observed across the dose range. Additionally, confirmed clinical responses were observed in patients with HGOC at the 60 mg BID dose.

As these data are from a small, non‐randomized, DE portion of a phase 1/2 study in Chinese female patients, the generalization of these results is limited. However, as these data confirmed the RP2D of pamiparib, they may serve as a foundation for future studies in this patient population. Once the results from the phase 2 dose‐expansion portion of this study as well as the results from the other studies of pamiparib monotherapy (NCT03712930, NCT03519230, and NCT03427814) and combination therapy (NCT03150810, NCT03150862, and NCT02660034) become available, more definitive conclusions can be drawn regarding the efficacy, safety, and tolerability of pamiparib across different solid tumors.

## CONFLICT OF INTEREST

Mei Dong, Yongmei Yin, Yan Song, Wei Li, Xiang Huang, Tongshan Wang, and Jing He have nothing to disclose. Binghe Xu reports receiving support for Speakers’ Bureau from AstraZeneca, Pfizer, Roche, Eisai, Novartis. Miao Li, Xiyan Mu, Li Li, Song Mu, and Wa Zhang are employees of BeiGene.

## AUTHOR CONTRIBUTIONS

Binghe Xu: writing‐review and editing, conceptualization, investigation, resources, supervision, validation, and writing‐original draft. Mei Dong: writing‐review and editing, conceptualization, investigation, resources, supervision, and writing‐original draft. Yongmei Yin: writing‐review and editing, conceptualization, investigation, resources, supervision, and writing‐original draft. Yan Song: writing‐review and editing, investigation, resources, and writing‐original draft. Wei Li: writing‐review and editing, investigation, resources, and writing‐original draft. Xiang Huang: writing‐review and editing, investigation, resources, and writing‐original draft. Tongshan Wang: writing‐review and editing, investigation, resources, and writing‐original draft. Jing He: writing‐review and editing, investigation, resources, and writing‐original draft. Miao Li: writing‐review and editing, conceptualization, formal analysis, methodology, project administration, resources, supervision, validation, and writing‐original draft. Xiyan Mu: writing‐review and editing, conceptualization, methodology, resources, supervision, validation, and writing‐original draft. Li Li: writing‐review and editing, conceptualization, formal analysis, methodology, resources, validation, and writing‐original draft. Song Mu: writing‐review and editing, conceptualization, formal analysis, resources, validation, and writing‐original draft. Wa Zhang: writing‐review and editing, conceptualization, formal analysis, resources, validation, and writing‐original draft.

## PREVIOUS DATA PRESENTATIONS

These data, in part, have been previously presented at the 2018 annual meeting of the American Academy of Cancer Research and the 2019 annual congress of the Chinese Society of Clinical Oncology.

## Supporting information

Table S1‐S4

## Data Availability

Upon request, and subject to certain criteria, conditions, and exceptions, BeiGene will provide access to individual de‐identified participant data from BeiGene‐sponsored global interventional clinical studies conducted for medicines (1) for indications that have been approved or (2) in programs that have been terminated. BeiGene will also consider requests for the protocol, data dictionary, and statistical analysis plan. Data requests may be submitted to medicalinformation@beigene.com.
