# Supplementary material for: Pamiparib dose escalation in Chinese patients with non‐mucinous high‐grade ovarian cancer or advanced triple‐negative breast cancer
Source: Cancer Med. 2020 Oct 31;10(1):109–18. doi: 10.1002/cam4.3575 (PMC7826463; doi:10.1002/cam4.3575)
Supplement: Supplementary file 1 — Table S1‐S4 [file CAM4-10-109-s001.docx]

**Supplementary Tables**

**Table S1. Patient Disposition**

|  | **20 mg BID (n=4)** | **40 mg BID (n=4)** | **60 mg BID (n=7)** | **Total**  **(N=15)** |
| --- | --- | --- | --- | --- |
| **No. patients treated** | 4 (100.0) | 4 (100.0) | 7 (100.0) | 15 (100.0) |
| **Discontinued treatment** | 3 (75.0) | 4 (100.0) | 7 (100.0) | 14 (93.3) |
| Progressive disease | 2 (50.0) | 3 (75.0) | 3 (42.9) | 8 (53.3) |
| Adverse event | 0 | 0 | 2 (28.6) | 2 (13.3) |
| Patient withdrawal | 0 | 1 (25.0) | 0 | 1 (6.7) |
| Lost to follow-up | 0 | 0 | 1 (14.3) | 1 (6.7) |
| Investigator’s discretion | 1 (25.0) | 0 | 1 (14.3) | 2 (13.3) |
| **Remained on treatment** | 1 (25.0) | 0 | 0 | 1 (6.7) |
| **Discontinued study** | 0 | 1 (25.0) | 1 (14.3) | 2 (13.3) |
| Lost to follow-up | 0 | 0 | 1 (14.3) | 1 (6.7) |
| Other | 0 | 1 (25.0) | 0 | 1 (6.7) |
| **Completed study** | 3 (75.0) | 3 (75.0) | 6 (85.7) | 12 (80.0) |
| **Remained in study** | 1 (25.0) | 0 | 0 | 1 (6.7) |

Abbreviation: BID, twice daily.

**Table S2. Dose Proportionality (PK Analysis Population)**

| **Parameter** | **n** | **Intercept (95% CI)** | **Slope (95% CI)** |
| --- | --- | --- | --- |
| AUC_0-inf_ (h*ng/mL) | 13 | 6.55 (3.81, 9.28) | 0.90 (0.16, 1.64) |
| AUC_last, ss_ (h*ng/mL) | 13 | 5.05 (2.81, 7.28) | 1.43 (0.82, 2.04) |
| C_max_ (ng/mL) | 15 | 3.43 (2.37, 4.48) | 1.06 (0.78, 1.34) |
| C_max, ss_ (ng/mL) | 13 | 3.01 (0.78, 5.23) | 1.42 (0.81, 2.03) |

Abbreviations: AUC, area under the plasma concentration-time curve from time of drug administration to last dose (steady state) or to infinity; CI, confidence interval; C_max_, maximum observed plasma concentration; inf, infinity; PK, pharmacokinetics; ss, steady state.

**Table S3. Disease Response Based on CA-125 Level (CA-125-Evaluable Population)**

|  | **HGOC**  **(n=8)** |
| --- | --- |
| **CA-125 response rate, n (%)** | **2 (25.0)** |
| Confirmed complete response | 1 (12.5) |
| Confirmed partial response | 1 (12.5) |
| **Time to response, months** | |
| n | 2 |
| Median (range) | 1.45 (1.4–1.5) |

Abbreviations: CA-125, carcinoma antigen 125; HGOC, high-grade ovarian cancer.

**Table S4. Summary of Progression-Free Survival (Safety Population)**

|  | **HGOC (n=9)** | **TNBC (n=6)** | **Total (N=15)** |
| --- | --- | --- | --- |
| **Events, n (%)** | **2 (22.2)** | **5 (83.3)** | **7 (46.7)** |
| Disease progression | 2 (22.2) | 5 (83.3) | 7 (46.7) |
| **Censored, n (%)** | **7 (77.8)** | **1 (16.7)** | **8 (53.3)** |
| New anticancer therapy | 1 (11.1) | 0 | 1 (6.7) |
| No disease progression or death | 5 (55.6) | 0 | 5 (33.3) |
| No post-baseline assessment | 1 (11.1) | 1 (16.7) | 2 (13.3) |
| **Median follow-up time, months (95% CI)** | **5.6 (0.03–31.84)** | **NE (0.03–NE)** | **6.3 (1.41–31.84)** |
| **Median PFS, months (95% CI)** | **NE (2.86–NE)** | **1.5 (1.45–1.61)** | **4.2 (1.48–NE)** |
| **Event-free rate at** | | | |
| 3 months (95% CI) | 85.7 (33.41–97.86) | 0 (NE–NE) | 50.0 (20.85–73.61) |
| 6 months (95% CI) | 68.6 (21.28–91.21) | 0 (NE–NE) | 40.0 (13.52–65.73) |
| 9 months (95% CI) | 68.6 (21.28–91.21) | 0 (NE–NE) | 40.0 (13.52–65.73) |

Abbreviations: CI, confidence interval; HGOC, high-grade ovarian cancer; NE, not estimable; PFS, progression-free survival; TNBC, triple-negative breast cancer.
